# Nerve Targeting via Myelin Protein Zero and the Impact of Dimerization on Binding Affinity

**DOI:** 10.3390/molecules27249015

**Published:** 2022-12-17

**Authors:** Nataliia Berehova, Tessa Buckle, Maarten P. van Meerbeek, Anton Bunschoten, Aldrik H. Velders, Fijs W. B. van Leeuwen

**Affiliations:** 1 Interventional Molecular Imaging Laboratory, Department of Radiology, Leiden University Medical Center, Albinusdreef 2, 2300 RC Leiden, The Netherlands; 2 Laboratory of BioNanoTechnology, Wageningen University & Research, Bornse Weilanden 9, 6708 WG Wageningen, The Netherlands

**Keywords:** fluorescence imaging, nerve targeting, myelin protein zero, fluorescence-guided surgery

## Abstract

Background: Surgically induced nerve damage is a common but debilitating side effect. By developing tracers that specifically target the most abundant protein in peripheral myelin, namely myelin protein zero (P0), we intend to support fluorescence-guided nerve-sparing surgery. To that end, we aimed to develop a dimeric tracer that shows a superior affinity for P0. Methods: Following truncation of homotypic P0 protein-based peptide sequences and fluorescence labeling, the lead compound Cy5-P0_101–125_ was selected. Using a bifunctional fluorescent dye, the dimeric Cy5-(P0_101–125_)_2_ was created. Assessment of the performance of the mono- and bi-labeled compounds was based on (photo)physical evaluation. This was followed by in vitro assessment in P0 expressing Schwannoma cell cultures by means of fluorescence confocal imaging (specificity, location of binding) and flow cytometry (binding affinity; K_D_). Results: Dimerization resulted in a 1.5-fold increase in affinity compared to the mono-labeled counterpart (70.3 +/− 10.0 nM vs. 104.9 +/− 16.7 nM; *p* = 0.003) which resulted in a 4-fold increase in staining efficiency in P0 expressing Schwannoma cells. Presence of two targeting vectors also improves a pharmacokinetics of labeled compounds by lowering serum binding and optical stability by preventing dye stacking. Conclusions: Dimerization of the nerve-targeting peptide P0_101–125_ proves a valid strategy to improve P0 targeting.

## 1. Introduction

Accidental surgical damage to nerves can yield debilitating side effects. For example, damage to key sensory nerves (6–22 mm in diameter) during orthopedic surgery may mean limbs lose function [1]. Alternatively, damage caused to small nerves (1–2 mm in diameter) during pelvic surgery can induce incontinence and erectile dysfunction [2,3,4]. Even when nerve sparing is specifically intended in prostate cancer patients, 30% still suffer from the effects of surgically induced damage [5,6]. Prevention of damage starts with the surgeon’s ability to identify nerves residing in the surgical field.

**Figure 1:**
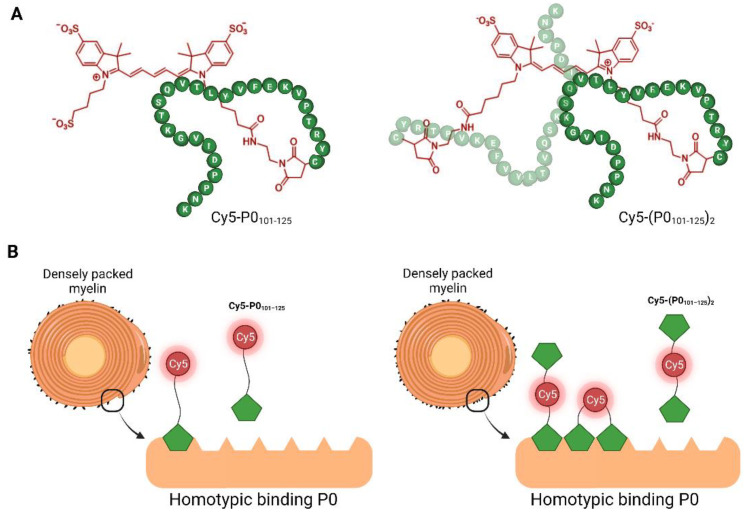
(**A**) Schematic structure of mono-labeled Cy5-P0_101–125_ and bi-labeled Cy5-(P0_101–125_)_2_ agents; (**B**) dimerization effect on binding affinity of Cy5-P0_101–125_ (left) and Cy5-(P0_101–125_)_2_ (right) to Schwann cells.

Nerve-specific fluorescence imaging [7] could help increase the specificity and sensitivity of intraoperative nerve identification. To this end different strategies for imaging have been put forward. In general, nerve imaging can be realized via neuronal tracing [8], by targeting the intracellular myelin basic protein (MBP) [9,10] or extracellular myelin protein zero (MPZ or P0) [11,12]. The latter especially provides an interesting imaging target, as it makes up 80% of the myelin protein content (Figure 1A, [11]) and is specific for the peripheral nervous system (PNS). Previous efforts towards the truncation of the extracellular portion of P0, followed by fluorescence labeling, allowed for P0 targeting in vitro and in vivo (mice and porcine models; lead Cy5-P0_101–125_) [11].

It is becoming increasingly obvious that signal intensity and signal-to-background ratios play a key role in fluorescent-guided surgery approaches [13]. Increasing the targeting affinity and via that route and enhancing the uptake of the agent is a well-accepted route for improving the signal intensity. A known strategy for affinity enhancement is the exploration of the multivalency concept. This concept is widely studied in the field of supramolecular chemistry [14,15,16]. Multiple studies also indicate that the multivalency approach also applies to imaging agents [17,18,19,20,21,22]. At the same time, there is evidence that multimerization of targeting vectors helps to “shield” the imaging agent (fluorescent dye) from the environment, hereby improving the pharmacokinetic properties [23,24,25].

We reasoned that the lead P0_101–125_ peptide would allow us to synthesize a dimeric Cy5-(P0_101–125_)_2_ agent with lower serum binding, dye stacking, and superior P0 affinity compared to the monomer, and with that, enhance the signal intensity. Bifunctional constructs containing two thiol-reactive maleimide groups formed the basis of the design for the peptide conjugation [26] (Figure 1).

Following synthesis, compounds were evaluated using NMR and MS. Chemical and photophysical properties were measured and analyzed. Fluorescence intensity and affinity enhancement of the bi-labeled agent were studied in vitro (flow cytometry and confocal microscopy) using cultures of P0-expressing Schwannoma cells compared to the parental mono-labeled Cy5-P0_101–125_.

## 2. Results

### 2.1. Chemistry

**Scheme 1:**
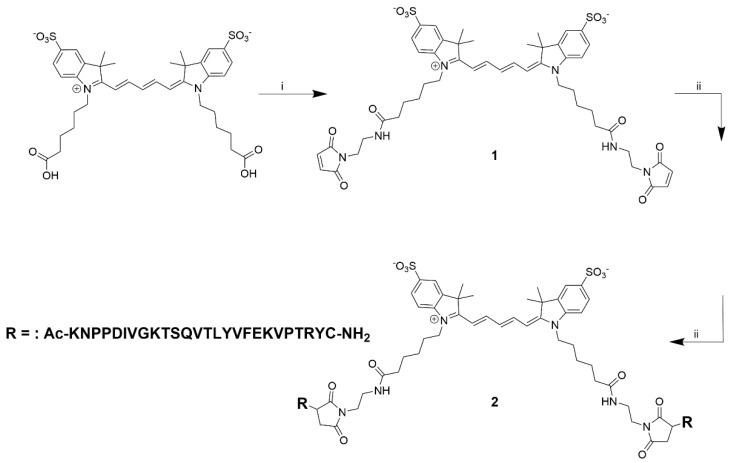
Reagents and conditions: (i) *N*-(2-aminoethyl)maleimide trifluoroacetate salt, PyBOP, *N*-methyl morpholine, DMF, r.t., 2 h; (ii) P0_101–125_, phosphate-buffered saline (pH 7,4), DMF, r.t., 2 h.

Following a literature procedure [27], we first synthesized COOH-(SO_3_)Cy5(SO_3_)-COOH. For the maleimide incorporation, the symmetrical COOH-(SO_3_)Cy5(SO_3_)-COOH was treated with *N*-(2-aminoethyl)maleimide trifluoroacetate salt, *N*-methyl morpholine was used as a base, and PyBOP was used as a coupling agent. Next, Cy5-(P0_101–125_)_2_ (2) was synthetized by adding excess of the free peptide P0_101–125_ to Maleimide-(SO_3_)Cy5(SO_3_)-Maleimide (1) in PBS (Scheme 1).

### 2.2. Chemical and Photophysical Properties

Table 1 provides a summary of the chemical and photophysical properties of the protein-conjugated compounds and the corresponding free dyes.

When compared to Cy5-P0_101–125_, the lipophilicity of Cy5-(P0_101–125_)_2_ was shown to be increased, while the degree of serum protein binding decreased by 26% (*p* = 0.0002). The glutathione stability tests only showed 4% degradation for both compounds.

**Figure 2:**
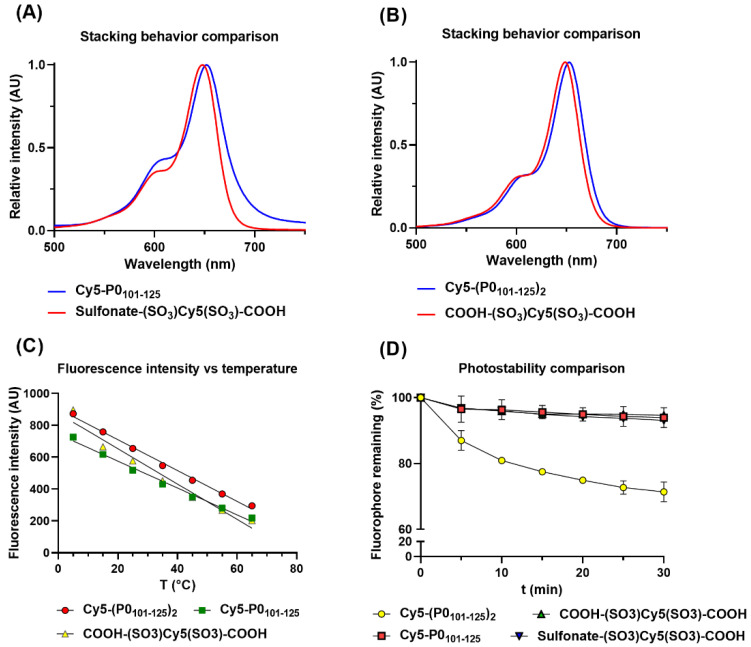
Photophysical properties of Cy5-P0_101–125_ and Cy5-(P0_101–125_)_2_. (**A**) Comparison in stacking behavior between Cy5-P0_101–125_ and corresponding free sulfonate-(SO3)Cy5((SO3)-COOH dye at 8mM measured in water. (**B**) Comparison in stacking behavior between Cy5-(P0_101–125_)_2_ and corresponding free COOH-(SO_3_)Cy5(SO_3_)-COOH dye at 8 mM measured in water; each spectrum was normalized at its wavelength of maximum absorbance. (**C**) Fluorescence intensity dependence of the temperature for Cy5-P0_101–125_, Cy5-(P0_101–125_)_2_ and symmetrical free dye COOH-(SO3)Cy5((SO3)-COOH; a trendline is included for all compounds. (D) The optical stability of Cy5-(P0_101–125_)_2_, Cy5-P0_101–125_, and of corresponding free dyes determined over the course of 30 min (means were fitted).

Brightness values for Cy5-P0_101–125_ and Cy5-(P0_101–125_)_2_ were comparable (Table 1: *p* = 0.3450). On the other hand, Cy5-(P0_101–125_)_2_ showed a slightly lower photostability than its monosubstituted counterpart (Figure 2D; *p* = 0.0139) as well as compared with free dyes (Table 1).

The stacking behavior of Cy5-(P0_101–125_)_2_ proved to be similar to that of the free dye COOH-(SO_3_)Cy5(SO_3_)-COOH at the same concentration (Figure 2B). Conversely, mono-labeled Cy5-P0_101–125_ yielded a higher “shoulder peak”, which is indicative for dye stacking [28].

Increase in temperature, with a resulting enhancement of the structural mobility in the bridging Cy5 dye, yielded a 65% loss of fluorescence intensity for a dimer and slightly more for a monomer (70%), while for the symmetrical free dye COOH-(SO_3_)Cy5(SO_3_)-COOH a loss in intensity of about 80% was seen (Figure 2C).

### 2.3. P0 Binding Affinity and Cellular Fluorescence Intensity

RT4-D12 Schwannoma cells were selected for in vitro experiments based on their origin and their specific P0-expressing properties [11].

**Figure 3:**
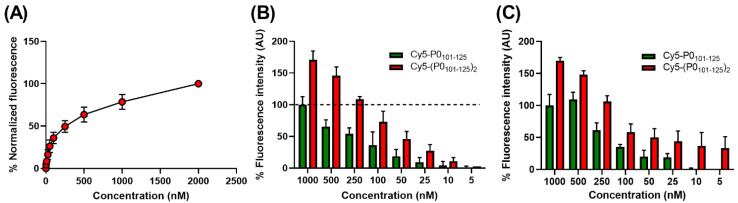
Quantified assessment of binding affinity and signal intensity. (**A**) Saturation binding curve for Cy5-(P0_101–125_)_2_ as determined using flow cytometry after incubation of cells with increasing tracer concentrations (0–2000 nM). (**B**) Quantification via flow cytometry and (**C**) via fluorescence confocal microscopy of the fluorescence intensity per cell for Cy5-P0_101–125_ (green bars) and Cy5-(P0_101–125_)_2_ (red bars) at different tracer concentrations.

Saturation binding experiments revealed a K_D_ for Cy5-(P0_101–125_)_2_ of 70.3 +/− 10 nM (Figure 3A), which is a 1.5-fold increase compared to Cy5-P0_101–125_ (K_D_: 104.9 +/− 16.7 nM; *p* = 0.003). Nonspecific binding was comparable for both tracers (0.020 for Cy5-P0_101–125_ and 0.022 for Cy5-(P0_101–125_)_2_. Quantification of the fluorescence intensity in flow-cytometry studies performed with a tracer concentration range between 5 and 1000 nM demonstrated a concentration-dependent signal intensity for both tracers (Figure 3B). On average, at the same concentration, a 44% higher (range 34–59%; Figure 3B) fluorescence signal intensity was seen for Cy5-(P0_101–125_)_2_ compared to the fluorescence signal intensity of Cy5-P0_101–125_ (*p* < 0.00001). As a result, the bench mark signal intensity for Cy5-P0_101–125_ at 1 mM [11] was comparable to the signal intensity seen for Cy5-(P0_101–125_)_2_, between 250–100 nM (Figure 3B), yielding about a 4-fold increase in staining efficiency.

**Figure 4:**
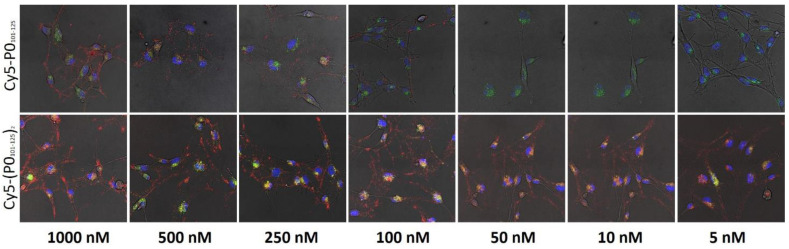
In vitro comparison of Cy5-P0_101–125_ and Cy5-(P0_101–125_)_2_. Fluorescence confocal imaging of P0-expressing RT4-D12 Schwannoma cells after incubation with different concentrations of either Cy5-P0_101–125_ or Cy5-(P0_101–125_)_2_ (5–1000 nM). P0 tracer in red, lysosomes in green, and nucleus in blue.

Fluorescence confocal microscopy of cultured Schwannoma cells confirmed P0 targeting and underscores the concentration-dependent staining observed with flow cytometry (Figure 3C and Figure 4), where cells could be clearly discriminated based on their P0 expression at using 100nM Cy5-(P0_101–125_)_2_. Only a faint P0-related signal could be detected after incubation of Cy5-P0_101–125_ at the same concentration. Cy5-(P0_101–125_)_2_ allowed effective visualization of P0 expression at a concentration as low as 10nM, and the signal could be detected down to 5 nM.

## 3. Discussion

By synthesizing Cy5-(P0_101–125_)_2_, we were able to create a dimeric imaging agent that has a superior affinity for P0 and more effectively stains cellular P0, thereby taking yet another step forwards in the realization of successful intraoperative nerve illumination.

As stated in the introduction, multimerization is commonly employed to increase binding affinity [18,29]. While there was no discernible difference in nonspecific binding for mono- and bi-protein-labeled compounds, we found that the presence of two targeting vectors on a single tracer boosted the K_D_ to 70.3 +/− 10.0 nM, converting to a 1.5-fold gain in receptor affinity compared to Cy5-P0_101–125_. This is a rise that is in line with previous reports mentioning peptide multimers [24,29]. Having an additional P0_101–125_ moiety also shields the Cy5 dye from serum interactions (Table 1), thereby lowering the serum binding by more than 20%. As serum binding of imaging agents makes for a notorious cause of background staining [30] and drives hepatic uptake/clearance [31], such a reduction supports more favorable pharmacokinetics. Finally, we found that the dimerization improved the optical properties of the nerve tracer by preventing dye stacking (see Figure 2A,B).

While comparing concentration dependance on stacking behavior of both protein-conjugated compounds (Figure S4A,B) we observed that a typical “stacking shoulder” appears only for a mono-labeled Cy5-P0_101–125_ at higher concentration. This indicates that P0_101–125_ moieties in the mono-substituted compound, at higher concentrations, aggregate with other monomers, thus bringing dyes in close vicinity of each other and causing a typical “dye-dye stacking” peak to occur in the absorbance spectrum (see Figure 2A) [27]. Conversely, addition of a second P0_101–125_ prevents such interactions from occurring.

Overall, Cy5-(P0_101–125_)_2_ was able to effectively stain cellular P0 at 4-fold lower concentrations compared to the mono-substituted compound, meaning it was effective at a lower dye-to-peptide ratio.

Despite the potential clinical benefit of nerve imaging, it has proved challenging to create nerve-specific imaging tracers. While various preclinical studies show promising results [32], there are a number of risks holding back the translation of these approaches. Hereby, toxicity is a major concern. Specifically, long-term toxic effects of nerve targeting tracers could ultimately do more harm than good, meaning the resulting complications could be worse that the complications that are avoided by providing a nerve-sparing surgical procedure. Using P0_101–125_ has as advantage that the central nervous system is not affected [11], and one can argue that the potential dose reduction (see Figure 3 and Figure 4) also helps reduce the chance of toxic effects, especially when combined with local injections [33]. More extensive in vivo studies are, however, needed to corroborate this.

## 4. Materials and Methods

All chemicals were received from Actu-All Chemical (Oss, The Netherlands), Sigma Aldrich (St. Louis, MO, USA), Tokyo Chemical Industry (Tokyo, Japan), Biosolve BV (Valkenswaard, The Netherlands), VWR Chemicals (Solon, OH, USA) and used without further purification. DMF and DMSO were dried over four Å molecular sieves at least for 24 h prior to use. Preparative high-pressure liquid chromatography (prep-HPLC) was performed on a Waters HPLC system (Waters Chromatography B.V., Etten-Leur, The Netherlands) using a 2545 quaternary gradient module pump and a 2489 UV detector. Dr. Maisch ReproSil-Pur 120 C18-AQ 10 μM (250 mm × 20 mm) column (Dr. Maisch HPLC GmbH, Ammerbuch-Entringen, Germany) was applied operating flow rate of 12 mL/min. HPLC analysis was carried out on Waters Alliance 2690 using a Waters symmetry C_18_ column (2.1 × 150 mm) with a gradient of 0.1% TFA in H_2_O/MeCN 95:5 to 0.1% TFA in H_2_O/MeCN 5:95 in 17 min (0.3 mL/min). Mass spectrometry was performed on a Microflex LT/SH MALDI-TOF mass spectrometer (Bruker, Billerica, MA, USA). Lyophilization was performed with a VaCo 2-II lyophilizer (Zirbus technology GmbH, Bad Grund (Harz), Germany). Absorption spectrometry was performed using a UV1280 UV–vis spectrometer (Shimadzu, Kyoto, Japan), and fluorescence spectrometry was performed using an LS55 (Perkin Elmer, Waltham, MA, USA). The fluorescence spectra at different temperatures were measured on an Agilent Cary Eclipse (Agilent Cary Eclipse, Santa Clara, CA, USA). NMR spectra were measured on a Bruker Avance 300 (Bruker, Billerica, MA, USA) operating at 300 MHz for 1 H and 75.00 MHz for 13 C, using the residual solvents (CDCl_3_ and DMSO-d6) as internal references, and on a Bruker Advance III spectrometer operating at 600 MHz for 1H and equipped with a 2.5 mm PH SEI probe (Bruker, Billerica, MA, USA). For photostability, a prototype D-Light P surgical laparoscope (KARL STORZ, Tuttlingen, Germany) was used. Fluorescence confocal imaging was performed using a SP8 WLL confocal microscope (Leica Microsystems, Wetzlar, Germany). Obtained images were analyzed using Leica Confocal Software (Leica Microsystems). Mean fluorescence intensity values per sample were measured using a LSRII flow cytometer (BD Biosciences, Franklin Lakes, NJ, USA) with APC-A settings (635 nm laser and 750 nm long-pass filter). The binding constant (K_D_) of the normalized geometric means were fitted with equations in the Prism 5 software (GraphPad, San Diego, CA, USA). Statistical evaluation (Student’s *t*-test) for all properties was performed in the Prism 5 software. Figure 1 was created using BioRender (https://biorender.com/, accessed on 7 October 2022).

### 4.1. Synthetic Procedures

Peptide P0_101–125_ (Ac-KNPPDIVGKTSQVTLYVFEKVPTRYC-NH_2_), Sulfonate-(SO_3_)Cy5(SO_3_)-Maleimide, and Cy5-P0_101–125_ were synthesized following a procedure previously reported by Buckle et al. [11].

Maleimide-(SO_3_)Cy5(SO_3_)-Maleimide (1) and Cy5-(P0_101–125_)_2_ (2) were synthesized by slightly adapting procedures reported before in the literature [11].

Synthesis of 1. First, 46 mg of HOOC-(SO_3_)Cy5(SO_3_)-COOH [27] (62 µmol) was dissolved in DMF (3 mL), followed by addition of *N*-(2-aminoethyl)maleimide trifluoroacetate salt (39 mg, 155 µmol) and PyBOP (12.9 mg, 24.7 µmol). To this mixture, *N*-methyl morpholine (54.5 µL, 495 µmol) was added, and the reaction mixture was stirred at room temperature (r.t.) for 2 h. The reaction mixture was subsequently acidified with trifluoroacetic acid (TFA) (50 µL) and purified using preparative HPLC, employing a gradient of 5–95% acetonitrile over 51 min. Lyophilization of product-containing fractions yielded the title compound as a dark blue solid (40.3 mg, 40.78 µmol, 66 % isolated yield). MALDI-TOF [C_49_H_57_N_6_O_12_S_2_^2−^ + 2H]^+^ calculated 988.2, found 988.3. ^1^H NMR (300 MHz, MeOD) δ 8.30 (dd, *J* = 17.0, 9.0 Hz, 2 H), 7.89 (s, 3 H), 7.87 (d, *J* = 1.4 Hz, 1 H), 7.35 (t, *J* = 7.7 Hz, 2 H), 6.80 (s, 4 H), 6.68 (t, *J* = 12.4 Hz, 1 H), 6.36 (d, *J* = 13.7 Hz, 2 H), 4.13 (t, *J* = 7.0 Hz, 4 H), 3.60 (dd, *J* = 6.4, 4.5 Hz, 4 H), 3.35 (dd, *J* = 6.4, 4.6 Hz, 4 H), 2.13 (t, *J* = 7.2 Hz, 4 H), 1.81 (dt, *J* = 11.6, 5.9 Hz, 4 H), 1.75 (s, 12 H), 1.64 (dt, *J* = 14.7, 7.2 Hz, 5 H), 1.52–1.37 (m, 4H), 1.31 (t, *J* = 7.4 Hz, 1 H).^13^C NMR (75 MHz, MeOD) δ 174.81, 173.92, 171.17, 154.72, 143.53, 142.01, 141.24, 134.10, 126.68, 119.96, 110.32, 103.94, 53.41, 49.20, 49.17, 48.46, 48.17, 47.89, 47.61, 47.32, 47.04, 46.75, 43.66, 37.51, 37.10, 35.11, 26.71, 26.45, 25.83, 24.83.

Synthesis of 2. Maleimide-(SO_3_)Cy5(SO_3_)-Maleimide (1) (6 mg, 6 µmol) and P0_101–125_ (Ac-KNPPDIVGKTSQVTLYVFEKVPTRYC-NH_2_; 45 mg, 15 µmol) were dissolved in a mixture of phosphate-buffered saline (PBS) (5 mL; pH 7.4) and DMF (1 mL) and allowed to react at r.t. for 2 h. The reaction mixture was diluted with water and acetonitrile. The crude product was purified from side products of the reaction and excess of the free peptide by preparative HPLC employing a gradient of (25–95% acetonitrile in 30 min) to yield the title compound as a light blue solid (25.3 mg, 35.8 µmol, 67% isolated yield). A 58 µM stock in Milli-Q was prepared for use in further experiments. MALDI-TOF [C_325_H_493_N_74_O_92_S_4_^4−^ + Na]^+^; calculated 7035.2, found 7035.8.

### 4.2. Chemical Stability

For the stability towards glutathione, which is used as a measure of stability towards thiols and amines in vivo, 125 µL of a 2 mM solution of the compound in (0.1 M HEPES pH 7.4, pre-sparged with N_2_) was added to 125 µL of a 4 mM solution of reduced glutathione in 0.1M HEPES pH 7.4 (pre-sparged with N_2_). The solutions were mixed and then placed in a sample manager. A sample was analyzed at 37 degrees over 6 h in total to determine the amount of compound relative to t = 0.

### 4.3. Photophysical Properties

The molar extinction coefficient (ε), quantum yield (QY), brightness, lipophilicity, serum protein binding, photo- and chemical stability, and stacking behavior were determined according to a slight modification of the protocol put forward by Hensbergen et al. [34]. Sulfonate-(SO_3_)Cy5(SO_3_)-COOH [27] was measured and used as a control for QY.

To determine the effect that temperature (range 5 to 65 °C) has on the fluorescence intensity, samples with a maximum absorption of 0.1 AU. were used. Samples were stirred during the measurements and were left to equilibrate after the fluorescence spectrometer reached the right temperature.

To determine the stacking behavior of the mono- and bi-labeled compounds and their corresponding dyes, a dilution series of Cy5-(P0_101–125_)_2_ and Cy5-P0_101–125_ were made, consisting of the dye concentrations ranging from 8 μM to 0.5 μM.

### 4.4. Photostability

For photostability measurements, the cuvettes were sealed and illuminated for 5 min (30 min in total) by a modified Storz D-Light P fluorescence imaging system [11] at a distance of 50 mm in a dark environment. After each illumination cycle, the fluorescence intensity was determined.

### 4.5. Fluorescence Confocal Microscopy

RT4-D12 Schwannoma cells were trypsinized and seeded onto 35 mm culture dishes that contained a glass insert (MatTek co, Emu Plains, New South Wales, Australia) two days prior to the imaging experiment. Then, 0.5–1000 nM Cy5-P0_101–125_ or Cy5-(P0_101–125_)_2_ was added one hour prior to imaging (incubation at 4 °C; *n* = 3 per tracer). Peptide solutions were sonicated for 20 s prior to addition to prevent aggregation of the peptide in solution. Lysotracker (lysotracker green; 2 µL/mL DND-26, Thermo Fisher, Waltham, MA, USA) and Hoechst (33,342, 1 mg/mL, 1:500, Thermo Fisher) were added to the to provide staining of lysosomes and the nucleus, respectively.

Images were acquired following Cy5 excitation at 633 nm. Emission was collected between 650–700 nm. For visualization of the lysosomes in the cells, a 488 nm laser was used for excitation, while emission was collected between 500–550 nm. Hoechst was excited at 405 nm, and emission was collected between 410–460 nm. Fluorescence confocal images were acquired using a Leica SP8 WL at sequential settings and 63× magnification. Image analysis was performed using Leica Confocal Software (Leica Microsystems). Semi-quantitative analysis of the Cy5 signal intensity was performed using the quantification tool in Fiji software according to previously described methods [35,36].

### 4.6. Flow Cytometry

Flow cytometric analysis was performed using RT4-D12 Schwannoma cells after incubation with either Cy5-P0_101–125_ or Cy5-(P0_101–125_)_2_. For saturation binding experiments, Cy5-labelled peptides were added to the cell samples in a range of 0–2000 nM in 50 µL of 0.1% bovine serum albumin (BSA) in PBS, as previously described [11,17]. After 1 h of incubation at 4 °C, the cells were washed two times with 300 µL of 0.1% BSA in PBS and re-suspended in 300 µL of 0.1% BSA. Cells were gated on Forward Scatter and Side Scatter, and a minimum of 10,000 viable cells were analyzed per sample. All experiments were performed in triplicate.

For evaluation of the differences in signal intensity per concentration between Cy5-P0_101–125_ and Cy5-(P0_101–125_)_2_ (5–1000 nM), the mean signal intensity of Cy5-P0_101–125_ at 1000nM was set at 100%, and intensities measured at other tracer concentrations or for Cy5-(P0_101–125_)_2_ were calculated as % increase or decrease from this point. The K_D_ values of the evaluated peptides were calculated using the “Binding-Saturation, One site—Total” nonlinear regression equation, as described previously [17].

### 4.7. Statistical Evaluation

Statistical evaluation for comparison of the photophysical properties (brightness, photostability), chemical properties (lipophilicity), K_D_, and fluorescence signal intensity values were performed using unpaired Student’s *t*-test comparing the means between Cy5-P0_101–125_ and Cy5-(P0_101–125_)_2_, where Cy5-P0_101–125_ is a control group. Value *p* < 0.05 are considering as a significant.

Normalization of the data set for stacking behavior was performed using Equation (1):

(1) $$x\left(\mathrm{normalized}\right)=\frac{\left(x-x_{\min}\right)}{\mathrm{range}\;\mathrm{of}\;x}$$

where $\mathrm{range}\;\mathrm{of}\;x={\;x}_{\max}-x_{\min}$.

## 5. Conclusions

Our findings indicate that Cy5-(P0_101–125_)_2_ has a superior affinity for P0, which led to 4-fold increase in efficiency compared to Cy5-P0_101–125_. Therefore, dimerization provides a feasible means to advance P0-specific nerve imaging strategies.

## 6. Patents

European patent application No.16180535.3.

## Figures and Tables

**Table 1:** Chemo- and photophysical properties of mono-labeled Cy5-P0_101–125_ and bi- labeled Cy5-(P0_101–125_)_2_ and corresponding free cyanine dyes.

Property	Sulfonate-(SO_3_)Cy5(SO_3_)-COOH *	Cy5-P0_101–125_	COOH-(SO_3_)Cy5(SO_3_)-COOH	Cy5-(P0_101–125_)_2_
**λ_ex_/λ_Em_ in H_2_O/PBS (stokes shift, nm)**	648/664 (16)	650/671 (21)	648/667 (19)	654/671 (17)
**Brightness (M^−1^ cm^−1^ in PBS)**	53.000	55.660 ^†^	46.000	49.242 ^†^
**Serum protein binding, %**	49 ± 8	89 ± 2	70 ± 4	63 ± 3
**logP_(o/w)_**	−1.42 ± 0.00	−1.39 ± 0.09	−1.62 ± 0.01	−0.57 ± 0.01
**Net charge**	−2	0	−3	+3
**Glutathione stability (at 6 h), %**	95	92	95	96
**Photobleaching, %**	93	94	95	71

λ_ex_/λ_Em_, excitation and emission wavelengths, respectively; PBS, phosphate-buffered saline; * previously reported by Spa et al. [27]; ^†^ calculated using the molar extinction coefficient of the free dye.

## Data Availability

The data presented in this study are available on request from the corresponding author. The data are not publicly available due to patent restrictions.

## Supplementary Materials

The following supporting information can be downloaded at https://www.mdpi.com/article/10.3390/molecules27249015/s1, Figure S1: NMR characterization of maleimide-(SO_3_)Cy5(SO_3_)-maleimide (**1**). (A) ^1^H NMR spectra (300 MHz, MeOD); (B) ^13^C NMR spectra (75 MHz, MeOD); Figure S2: MALDI-TOF mass spectra of (A) maleimide-(SO_3_)Cy5(SO_3_)-maleimide (**1**) and (B) Cy5-(P0_101–125_)_2_ (2); Figure S3: ^1^H NMR spectra of (A) Cy5-P0_101–125_ (600 MHz, D_2_O), (B) Cy5-(P0_101–125_)_2_ (600 MHz, D_2_O), (C) Cy5-(P0_101–125_)_2_ (600 MHz, DMSO); Figure S4: Concentration-dependent stacking behavior of Cy5-P0_101–125_ and Cy5-(P0_101–125_)_2_ and corresponding free dyes measured in H_2_O.

## Sample Availability

Samples of the compounds Cy5-P0_101–125_ and Cy5-(P0_101–125_)_2_ (including non-functionalized peptides and dye components) are available from the authors.
