# Supplementary material for: Nerve Targeting via Myelin Protein Zero and the Impact of Dimerization on Binding Affinity
Source: Molecules. 2022 Dec 17;27(24):9015. doi: 10.3390/molecules27249015 (PMC9786614; doi:10.3390/molecules27249015)
Supplement: Supplementary file 1 [file molecules-27-09015-s001.zip › molecules-2043435-supplementary.pdf]

Supporting information for

Nerve Targeting via Myelin Protein Zero and the Impact of Dimerization on Binding Affinity

Nataliia Berehova [1], Tessa Buckle [1], Maarten P. van Meerbeek [1], Anton Bunschoten [2], Aldrik H. Velders [2], and Fijs W.B. van Leeuwen [1,2,] *

[1] Interventional Molecular Imaging Laboratory, Department of Radiology, Leiden University Medical Center, Albinusdreef 2, 2300 RC Leiden, The Netherlands
[2] Laboratory of BioNanoTechnology, Wageningen University & Research, Bornse Weilanden 9, 6708 WG Wageningen, The Netherlands
* Correspondence: f.w.b.van_leeuwen@lumc.nl

Contents:

Figure S1: NMR characterization of ***Maleimide-(SO$_3$)Cy5(SO$_3$)-Maleimide (1)***

Figure S2: MALDI-TOF mass spectra of ***Maleimide-(SO$_3$)Cy5(SO$_3$)-Maleimide*** *(**1**) and* **Cy5-(P0$_{101–125}$)$_2$** (**2**)

Figure S3: ^{1}H NMR spectra of **Cy5-P0$_{101–125}$** in D_2O and **Cy5-(P0$_{101–125}$)$_2$** in D_2O and DMSO

Figure S4: Concentration dependent stacking behavior of **Cy5-P0$_{101-125}$** and **Cy5-(P0$_{101-125}$)$_2$** and corresponding free dyes measured in H_2O.

(A)

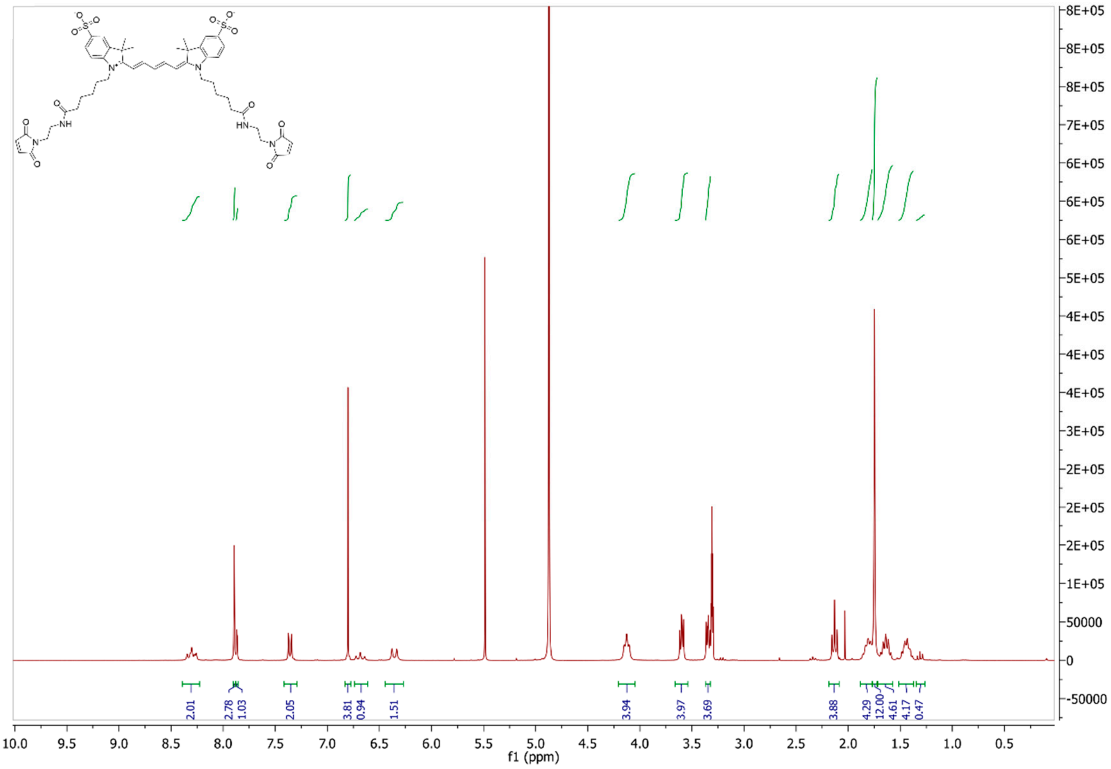

(B)

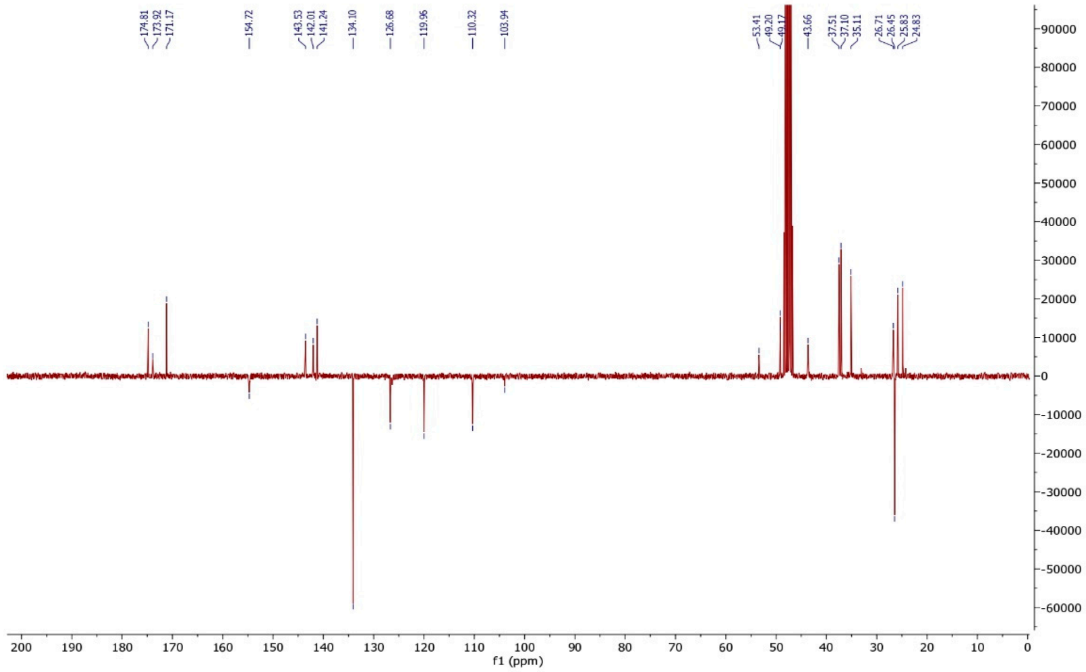

Figure S1: NMR characterization of *Maleimide-(SO_3)Cy5(SO_3)-Maleimide (1)*. (A) ^{1}H NMR spectra (300MHz, MeOD); (B) ^{13}C NMR spectra (75 MHz, MeOD).

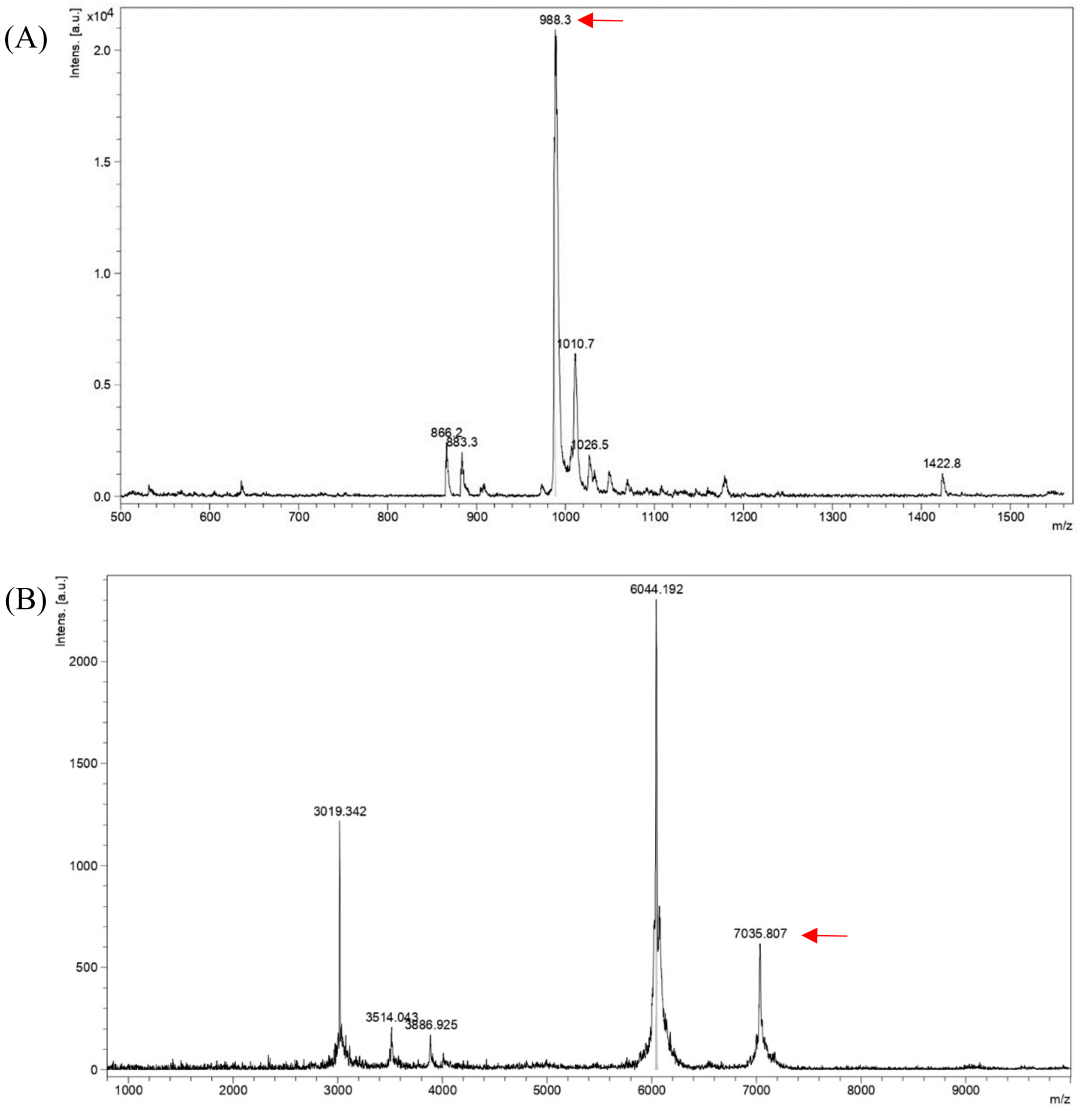

Figure S2: MALDI-TOF mass spectra of (A) *Maleimide-(SO_3)Cy5(SO_3)-Maleimide* - 988.3 (**1**) and (B) *Cy5-(P0$_{101–125}$)$_2$* – 7035,8 (**2**).

Others MS peaks in <u>Figure S2 (A)</u> can be contributed to: 1) side products : 866,2 - Maleimide-(SO_3)Cy5(SO_3)-COOH, 2) Maleimide-(SO_3)Cy5(SO_3)-Maleimide + salts : 1010,7 - Maleimide-(SO_3)Cy5(SO_3)-Maleimide and Na^+ and 1026,5 - Maleimide-(SO_3)Cy5(SO_3)-Maleimide and K^+, and 3) 1422.8 – matrix (reference peak).

Others MS peaks in <u>Figure S2 (B)</u>: 3019.3 – free P0$_{101-125}$ peptide peak; 6044,19 – mass of two P0$_{101-125}$ peptides.

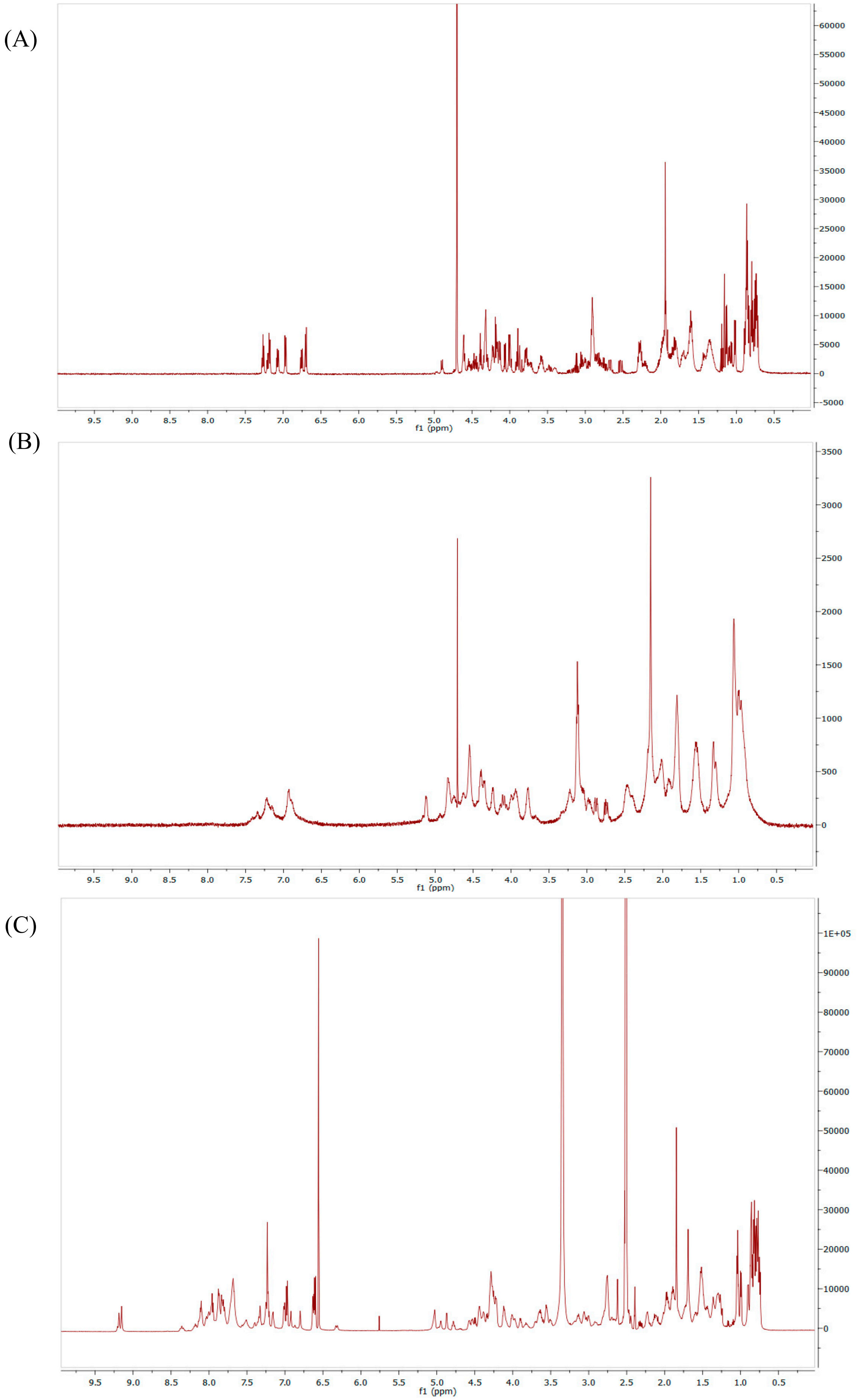

Figure S3. ^{1}H NMR spectra of (A**) Cy5-P0$_{101–125}$** (600MHz, D_2O), (B**) Cy5-(P0$_{101–125}$)$_2$** (600MHz, D_2O), (C) **Cy5-(P0$_{101–125}$)$_2$** (600MHz, DMSO).

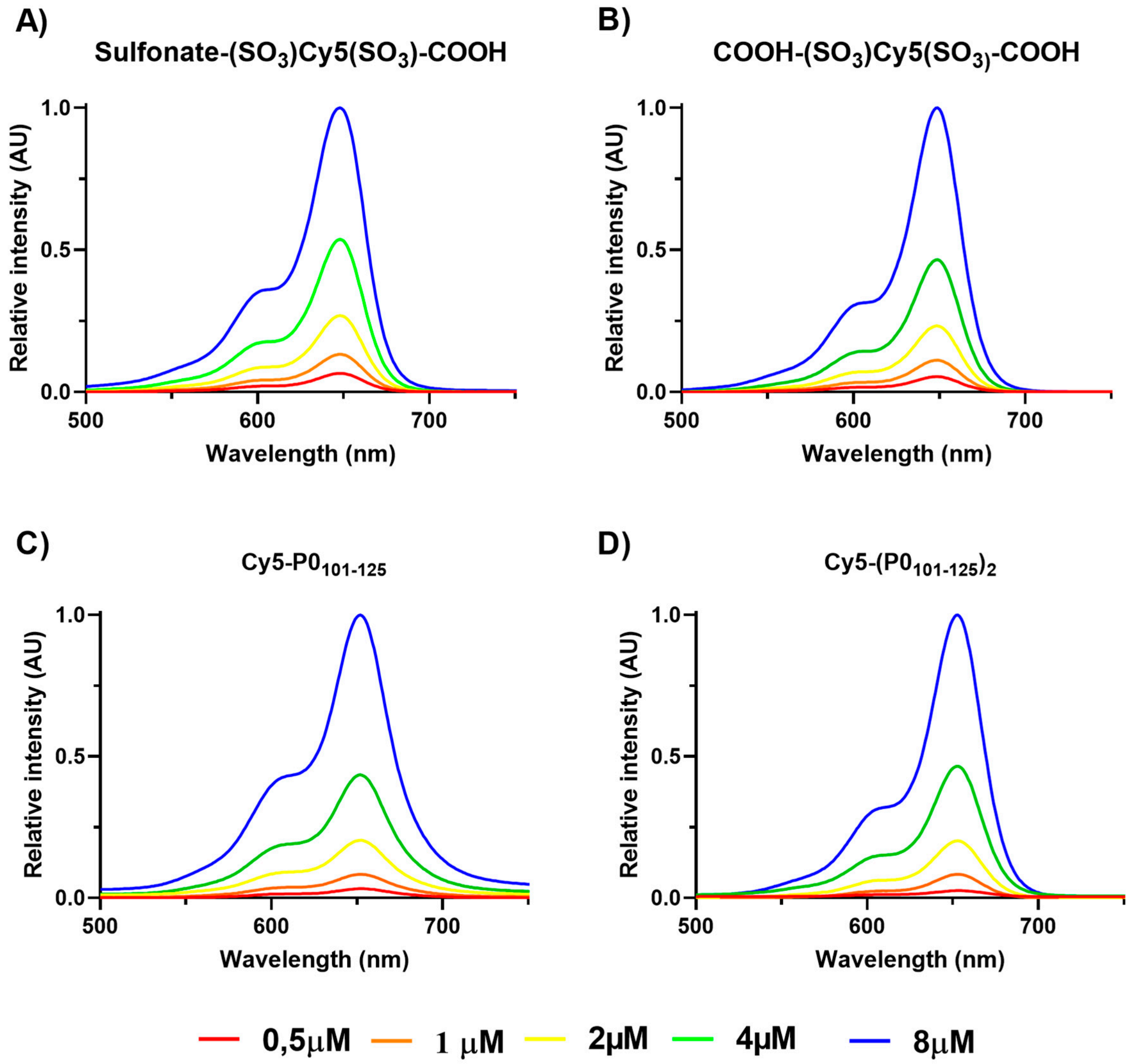

Figure S4. Concentration dependent stacking behavior of **Cy5-P0$_{101-125}$** and **Cy5-(P0$_{101-125}$)$_2$** and corresponding free dyes measured in H_2O.
